# 1368. Stakeholders Perceptions on Barriers and Facilitators to Opt-Out Infectious Diseases Care in Jails

**DOI:** 10.1093/ofid/ofac492.1197

**Published:** 2022-12-15

**Authors:** Emma M Smyth, Laura Lodolo, Yvane Ngassa, Curt Beckwith, Alysse Gail Wurcel

**Affiliations:** Tufts Medical Center, Boston, Massachusetts; Tufts University School of Medicine, Boston, Massachusetts; Tufts Medical Center, Boston, Massachusetts; Alpert Medical School of Brown University, Providence, Rhode Island; Tufts Medical Center, Boston, Massachusetts

## Abstract

**Background:**

Despite national guidelines on infectious disease testing and vaccination in prisons, there is heterogeneity in operationalization. Previous literature has shown that using the “opt-out approach” to infectious diseases testing increases equitable access. Although opt-out testing is endorsed by national guidelines, there is little research on the acceptability from key stakeholders in jail healthcare. Additionally, perspectives on using the opt-out approach to vaccination are unknown.

Interview Themes

**Figure:**
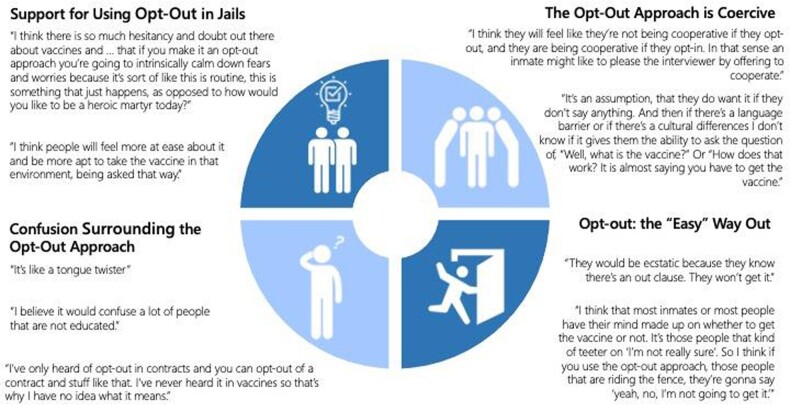

Themes and quotes that came out of semi-structured qualitative interviews.

**Methods:**

We developed an interview guide based on the Theoretic Domains Framework. Guided by the 7Ps framework for stakeholder engagement, we conducted semi-structured interviews with key stakeholders in jail healthcare, including people incarcerated in Hampden County jail, clinicians working in jail and community settings, corrections officers, and representatives from public health, government, and industry. All persons eligible to receive a stipend were offered $50 for participation. Interviews were offered in the jail in English and in Spanish.

**Results:**

Between July 2021—March 2022, 48/108 (44%) of people approached agreed to be interviewed including 13 incarcerated men. Most stakeholders agreed that infectious diseases testing and vaccination in jails are essential tools for mitigating the spread of disease. Major themes that emerged from stakeholder interviews included confusion about the wording of the “opt-out” approach, reluctance to operationalize “opt-out” testing because of concerns for coercion, and concern that it gives people any “easy out” from taking the test or vaccines. People who supported the operationalization of opt-out testing were more often stakeholders from the community, including public health experts, researchers, and clinicians.

**Conclusion:**

Although “opt-out” testing is evidence-based, we found that key stakeholders who work and are incarcerated in jails did not understand the concept and did not support implementation. Our work is further evidence that successful implementation of public health strategies requires vetting from all key stakeholders in jail healthcare, especially people working and incarcerated in jail when developing strategies to improve healthcare.

**Disclosures:**

**Curt Beckwith, MD**, Gilead Sciences, Inc: Grant/Research Support.

